# Global distribution modelling, invasion risk assessment and niche dynamics of *Leucanthemum vulgare* (Ox-eye Daisy) under climate change

**DOI:** 10.1038/s41598-019-47859-1

**Published:** 2019-08-06

**Authors:** Rameez Ahmad, Anzar A. Khuroo, Bipin Charles, Maroof Hamid, Irfan Rashid, N. A. Aravind

**Affiliations:** 1 Centre for Biodiversity & Taxonomy, Department of Botany, University of Kashmir, Srinagar, 190006 J & K India; 2 Ashoka Trust for Research in Ecology and the Environment (ATREE), Royal Enclave, Srirampura, Jakkur PO, Bengaluru, 560064 India; 3 Biological Invasions Laboratory, Department of Botany, University of Kashmir, Srinagar, 190006 J & K India

**Keywords:** Invasive species, Ecology

## Abstract

In an era of climate change, biological invasions by alien species represent one of the main anthropogenic drivers of global environmental change. The present study, using an ensemble modelling approach, has mapped current and future global distribution of the invasive *Leucanthemum vulgare* (Ox-eye Daisy) and predicted the invasion hotspots under climate change. The current potential distribution of Ox-eye Daisy coincides well with the actual distribution records, thereby indicating robustness of our model. The model predicted a global increase in the suitable habitat for the potential invasion of this species under climate change. Oceania was shown to be the high-risk region to the potential invasion of this species under both current and future climate change scenarios. The results revealed niche conservatism for Australia and Northern America, but contrastingly a niche shift for Africa, Asia, Oceania and Southern America. The global distribution modelling and risk assessment of Ox-eye Daisy has immediate implications in mitigating its invasion impacts under climate change, as well as predicting the global invasion hotspots and developing region-specific invasion management strategies. Interestingly, the contrasting patterns of niche dynamics shown by this invasive plant species provide novel insights towards disentangling the different operative mechanisms underlying the process of biological invasions at the global scale.

## Introduction

In an era of the climate change, biological invasions by invasive alien species represents one of the main anthropogenic drivers of global environmental change^1^. Invasive alien species pose a serious threat to biodiversity and natural resources^2^ including reductions in the native biota^1,3–5^, alteration of the ecosystem pools and fluxes^6^ and significant economic losses^7,8^. Aided by anthropogenic activities, global climate change is predicted to accelerate the expansion of invasive species outside of their native biogeographical regions^3,9^. Although, there is a complex relationship between biological invasions and climate change^10^, the impact of changing climate is becoming increasingly evident in rapid shifting of species’ geographical distribution, changing lifecycle, population dynamics and life history traits of invasive species^11,12^. Climatic factors are one of the main determinants of the overall distribution of invasive species due to their synergistic effects^4,13^. Recently, a number of studies have reported changing patterns in habitat suitability and range expansion of invasive species under the changing global climate^14–20^. Simultaneously, a few studies also predicted a range contraction in the presently suitable habitat of invasive species under future climatic conditions^14,21^.

**Figure 1:**
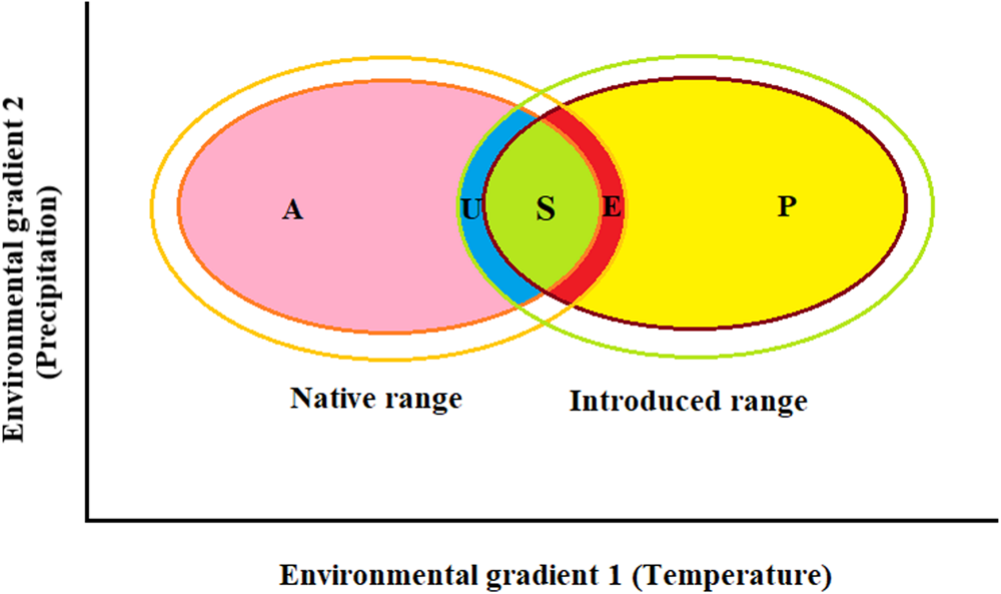
Conceptual framework of invasion niche dynamics: Schematic representation of different niche dynamic categories within the environmental space showing the distinction between niche expansion and pioneering and between niche unfilling and abandonment, following Atwater *et al*.^24^ and Guisan *et al*.^37^. Yellow and green circles represent the available climates and red and brown circles represent the occupied climates in case of native and introduced regions respectively. Different niche dynamic categories are indicated by solid colours viz: pink: abandonment (*A*) – proportion of the native niche not overlapping (non-intersecting) with the invaded niche outside the analogous environments; yellow: pioneering (P) - proportion of the introduced (invaded) niche not overlapping with the native niche outside the analogous environments; blue: unfilling (U): proportion of the native niche not overlapping with the invaded niche within the analogous environments; light green: stability (S) - proportion of the native niche overlapping with the invaded niche within the analogous environments and red: expansion (E) - proportion of the invaded niche not overlapping with the native niche within the analogous environments.

Biological invasions have also been studied from a niche conservatism perspective, i.e. the tendency of invasive species to retain similar niche in the native and introduced regions^22^ and niche shift perspective, i.e. any change in the niche position between the native and introduced regions^23,24^, or both^25^. Niche conservatism and shifts can have wide applications for understanding the effects of climate change on biological invasions^18,22,26^. Many research studies have reported niche shifts to be rare in the invaded regions of invasive alien species^27,28^; but some recent studies have reported the frequent occurrence of niche shifts among the invasive alien plants^24,29–31^. The reason behind these contrasting results mostly lie in conceptual differences associated with environmental availability^32^, as several studies have taken into consideration the nature of environment^28,33,34^, while others have not^29,35,36^. Therefore, a better understanding of the conceptual and methodological framework underlying invasion niche dynamics may enhance our ability in evaluating the true niche shifts^24,32,37^. More specifically the niche dynamics of invasive species comprises of five categories, with niche unfilling, stability and expansion occuring in analogous environmental conditions and abandonment and pioneering found in non-analogous environments (see Fig. 1 for detailed explanation).

In recent times, species distribution models (SDMs) have been widely used to predict distribution of invasive species based on niche conservatism at the global scale^13,38^. However, one major challenge with respect to these SDMs is that, currently a large number of modelling algorithms are available and increasing at a rapid pace too, thus making it difficult to select the most appropriate algorithm and suitable methodology^39^. Further, recent studies have also reported that the performance of each algorithm varies significantly, thereby, making the choice of the appropriate modelling alogarithm even more difficult. To overcome these limitations, there is an emerging scientific consensus to simultaneously apply several algorithms (e.g. ensemble modelling^40,41^) within a consensus modelling framework^42,43^. Such a technique of ensembling approach accounts for uncertainties in prediction of single algorithm by combining their predictions^44,45^, thereby increasing the predictive power of species distribution forecasts^43^. *Biomod2* package (https://CRAN.Rproject.org/package=biomod2)^46^ provides such a platform for ensemble forecasting within open-source R environment^47^. Besides it also utilizes a wide range of approaches to compare different modelling algorithms and to test the predictive power of these algorithms^48,49^.

**Figure 2:**
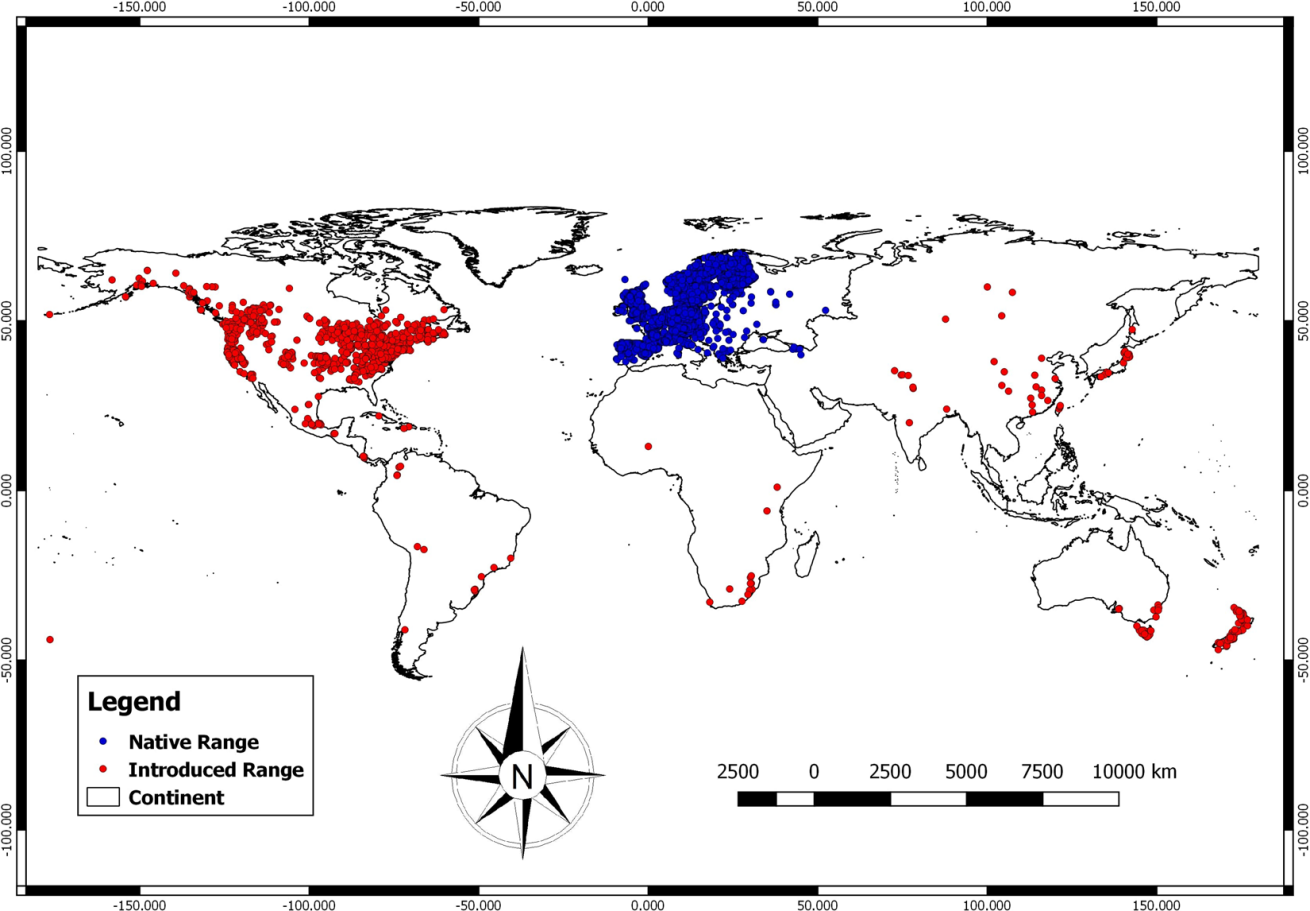
Global distribution map of *Leucanthemum vulgare*. (Blue dot represents native regions and red dot represents introduced regions).

Of the various invasive alien plant species across the world, Ox-eye Daisy, scientifically named as *Leucanthemum vulgare* Lam. (hereafter referred to as *Leucanthemum* in the present study) is a serious invasive weed native to Europe and Western Asia (Armenia)^2,50^ and has become naturalized and/or invasive in all the continents except Antarctica^2,51^ (Fig. 2). The species is a perennial herb belonging to family Asteraceae. In its introduced regions, it invades disturbed habitats like grazing pastures, open meadows, roadside areas and forest openings, thereby reducing the native plant species diversity and results in the formation of mono-specific stands^2,50,52^. Moreover, the species is a high altitudinal invasive species invading the subalpine mountainous regions across the globe, resulting in the invasion of natural ecosystems harbouring endemic species^2,52^.

To date, scientific research on *Leucanthemum* has mainly focused on its biology^51^ and ecological impacts^2,52^. To the best of our knowledge, studies on distribution modelling and impacts of climate change on this invasive species are still lacking. Therefore, modelling habitat suitability and testing its climatic niche conservatism under the future scenarios of climate change can provide robust tools for developing risk assessment protocol and long-term management strategies to prevent the introduction as well as to predict its future invasion hotspots^14,18,31^. The present study for the first time, using an ensemble modelling approach, has mapped the current and future potential global distribution of *Leucanthemum* and tested the niche conservatism of this invasive species between its native and introduced regions.

Specifically, the present study aimed to address the following research questions: (i) what is the potential global distribution of *Leucanthemum* under current and future climatic scenarios? (ii) which areas of the world are at the risk of its invasion under climate change, (i.e. to predict and map global invasion hotspots)? (iii) whether this invasive species undergoes shift in its climatic niche between native and introduced regions, and to disentangle the different dimensions of niche shift at regional scale?

## Results

### Model performance

**Figure 3:**
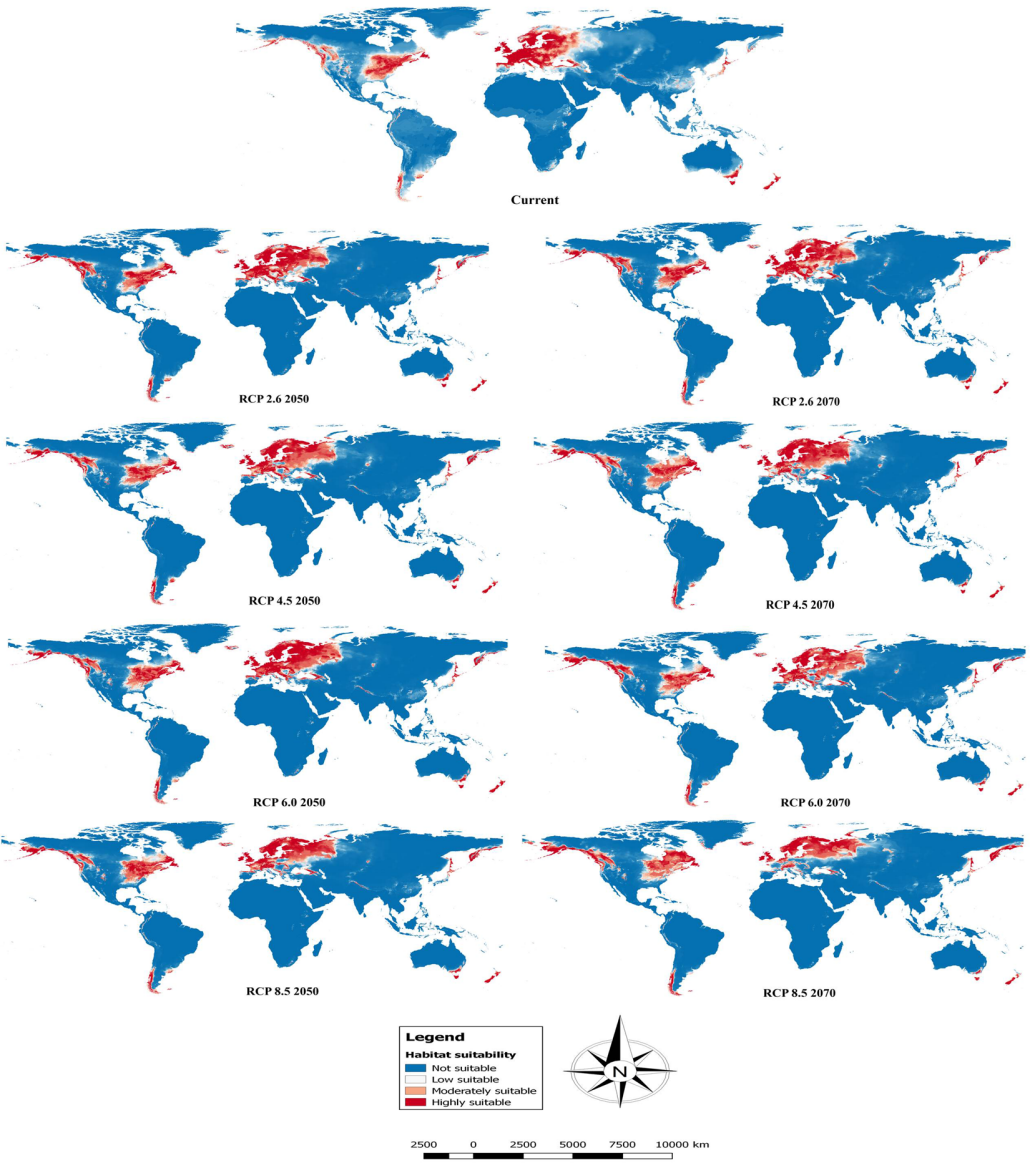
Ensemble maps showing the potential global distribution of *Leucanthemum vulgare* under current and future scenarios. Blue = Not suitable, White = Low suitable, Light red = Moderately suitable and Red = Highly suitable. The figure was generated using GIS software (QGIS v2.14.20, https://qgis.org/).

In the present study, the final ensemble model obtained by the combination of various models had area under the curve (AUC) value of 0.97, true skills statistics (TSS) value of 0.850 and Cohen’s KAPPA (KAPPA) value of 0.845 indicating that the model performed better in predicting the suitable habitat area for the species (Supplementary Table S1). The model predicted that the suitable habitat area for the target species will increase by the years 2050 and 2070 compared to the current period. Interestingly, many areas currently unsuitable will become increasingly suitable, while some currently suitable regions will become unsuitable in the future (Fig. 3).

### Current invasion pattern

The potential distribution map created by the ensemble model, based on the current climatic conditions and occurrence records of *Leucanthemum* showed that under the current climatic conditions, about 20,419,818 km^2^ (14.04%) of the total area across the globe is suitable for potential invasion by *Leucanthemum*. Of this total suitable area, 12,695,570 km^2^ (62.17%) possess high suitability, while as 3,951,795 km^2^ (19.35%) and 3,772,453 km^2^ (18.47%) have moderate and low suitability, respectively **(**Fig. 3, Table 1**)**.The current model showed that the regions having suitable and optimal environmental conditions for the invasion of the species are in the southern part of Alaska, eastern and south-western parts of Canada, parts of Mexico, the eastern part of United States of America and coastal parts of Columbia and Ecuador; the southern part of Chile and south-eastern part of Argentina in Southern America; almost the entire western part of Europe; the southern part of South Africa, the central part of Ethiopia and Kenya and northern part of Tanzania in Africa; parts of western Himalaya in India, Pakistan and Nepal, north-western and southern parts of China and Japan in Asia and the southern part of Australia and almost all of New Zealand in Australasia **(**Fig. 3**)**.

**Table 1:** Area in square kilometres (Km^2^) showing suitable habitats under current and future climatic scenarios for two time periods (2050 and 2070).

Range	Habitat Suitability	Current	RCP 2.6		RCP 4.5		RCP 6.0		RCP 8.5	
			2050	2070	2050	2070	2050	2070	2050	2070
**0**.**00–0**.**25**	Not Suitable	124,996,430	123,353,400 (−1.31%)	123,758,281 (−0.99%)	123,827,455 (−0.93%)	122,694,336 (−1.84%)	123,540,465 (−1.16%)	123,161,935 (−1.46%)	124,111,777 (−0.70%)	124,006,813 (−0.79%)
**0**.**25–0**.**50**	Low Suitable	3,772,453	3,690,911 (−2.16%)	3,377,305 (−10.47%)	3,766,096 (−0.16%)	3,573,694 (−5.26%)	3,352,930 (−11.12%)	3,800,403 (0.74%)	3,481,659 (−7.70%)	3,612,349 (−4.24%)
**0**.**50–0**.**75**	Moderately Suitable	3,951,795	4,479,280 (13.34%)	4,872,781 (23.30%)	4,642,564 (17.47%)	5,442,511 (37.72%)	4,264,561 (7.91%)	5,046,095 (27.69%)	4,391,528 (11.12%)	4,810,623 (21.73%)
**0**.**75–1**.**00**	Highly Suitable	12,695,570	13,892,643 (9.42%)	13,407,865 (5.61%)	13,180,117 (3.81%)	13,705,692 (7.95%)	14,258,277 (12.30%)	13,407,799 (5.61%)	13,431,269 (5.79%)	12,986,447 (2.29%)
**0**.**25–1**.**00**	Overall Suitable	20,419,818	22,062,834 (8.04%)	21,657,951 (6.06%)	21,588,777 (5.72%)	22,721,897 (11.27%)	21,875,768 (7.13%)	22,254,297 (8.98%)	21,304,456 (4.33%)	21,409,419 (4.84%)

### Future invasion risk

**Figure 4:**
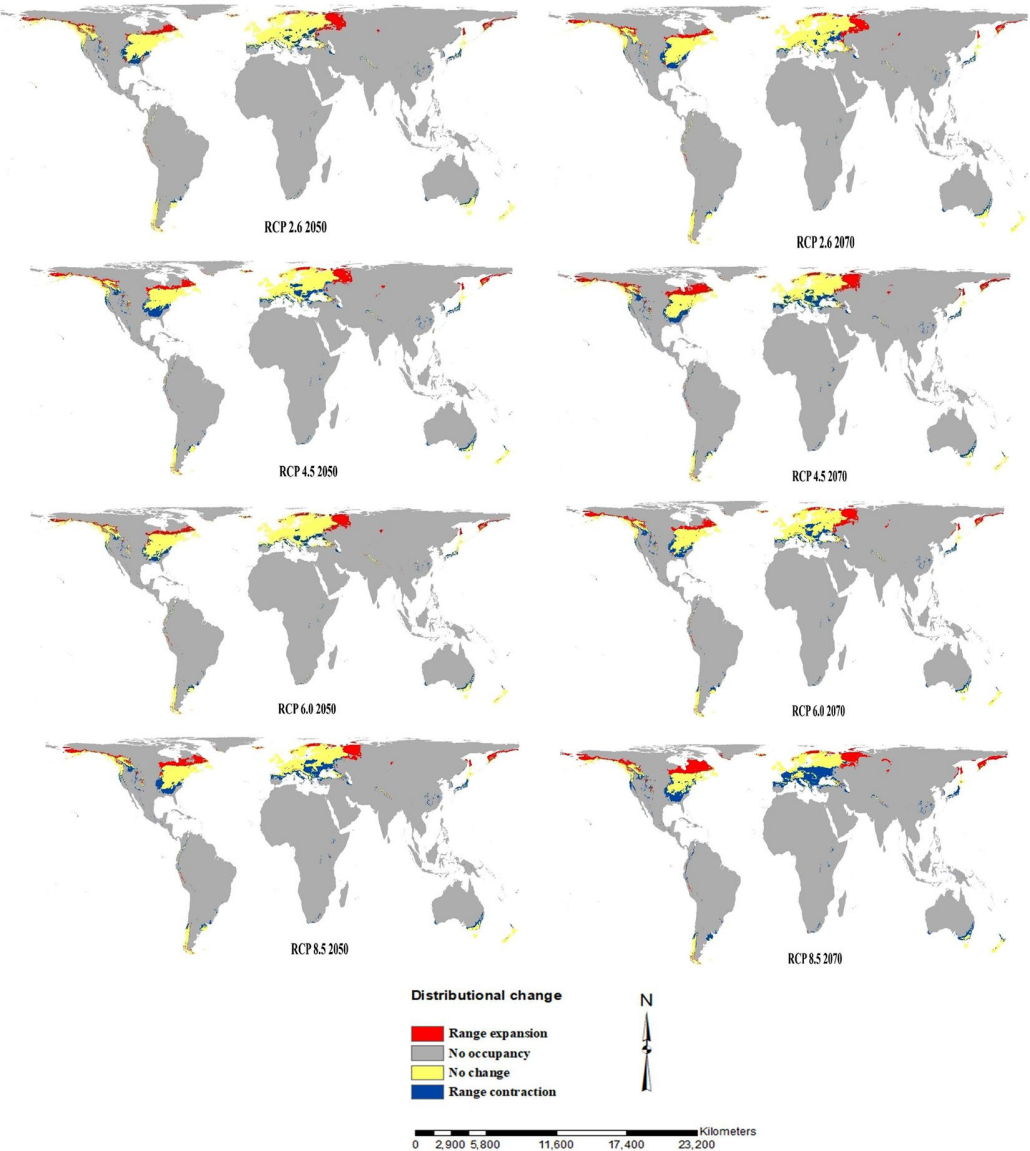
Distributional change maps between the two SDMs (current and future) showing future areas of change. Range expansion (red colour), no ocupancy (i.e. absent in both, grey colour), no change (i.e stable, yellow colour) and range contraction (blue colour)]. The figure was generated using ArcMap 10.2.2, http://desktop.arcgis.com/en/arcmap/.

The results of the ensemble model based on the future climatic scenarios (RCPs 2.6–8.5) for two time periods (2050 and 2070) predicted that the overall habitat suitability for potential invasion by the species will increase in all the RCP’s. The increasing future habitat suitability ranges between 21,304,456 km^2^ (with 4.33% increase as compared to current suitability) under RCP 8.5 2050 to 22,721,897 km^2^ (with 11.27% increase) under RCP 4.5 2070 **(**Table 1**)**. Our analysis also showed that both the moderately and highly suitable area for the potential invasion of the species increases in all the future climatic scenarios. The moderately suitable area increases between 4,264,561 km^2^ (7.91%) under RCP 6.0 2050 to 5,442,511 km^2^ (37.72%) under RCP 4.5 2070, while as highly suitable area increases from 12,986,447 km^2^ (2.29%) under RCP 8.5 2070 to 14,258,277 km^2^ (12.30%) under RCP 6.0 2050 **(**Table 1**)**. Under all the future RCP’s the regions showing future increase in moderately and highly suitable areas include southern Alaska, western and eastern Canada, western Peru, the southern part of Chile, central and north-eastern Norway and north-eastern, north-western and central parts of Russia (Fig. 4**)**. In contrast, some of the currently suitable regions show range contractions under the future climate *viz*. central, south-eastern and north-western parts of Canada, north-western Columbia, south-eastern Argentina, central and southern Europe, parts of western Himalaya, the southern part of South Africa, south-eastern China, parts of Japan and south-eastern Australia (Fig. 4). In short, although the species showed both range expansion and contraction under the future climatic scenarios, however the range expansion predictions exceeded the range contraction predictions. As a result, the overall suitable area as well as the areas of moderate and high habitat suitability for the potential invasion of the species increases over time under the climate change.

### Regional invasion risk assessment (current and future)

#### Current invasion risk assessment

Under the current climatic conditions, *Leucanthemum* is capable of invading 56% of the total area of Oceania, hence this is a high-risk region, whereas the other regions: Northern America, Australia, Southern America, Asia and Africa are categorised as low risk regions with 8%. 4%, 2%, 1% and 0.3% of their area capable of being invaded, respectively (Supplementary Table S2).

#### Future invasion risk assessment (2050 and 2070)

The results predicted that the Oceania will remain as a high-risk region for the potential future invasion of the species across all the RCP’s and for both the time periods (2050 and 2070) with the percentage risk ranging from 46% (under RCP 8.5 for 2070) to 62% (under RCP 2.6 for 2070). Northern America is predicted to be at moderate risk with percentage risk ranging from 11% (under RCP 2.6–2070 and RCP 4.5–2050) to 14% (under RCP 8.5 for 2070). The remaining regions: Southern America, Australia, Asia and Africa are all predicted to be at low risk with percentage risk values of 2 to 3%, 2 to 4%, 1 to 2% and 0.01 to 0.04%, respectively (Supplementary Tables S3–S10). In short, Oceania and Northern America remain as the invasion hotspots for this invasive species, both currently and under future scenarios of climate change.

#### Niche conservatism test

**Figure 5:**
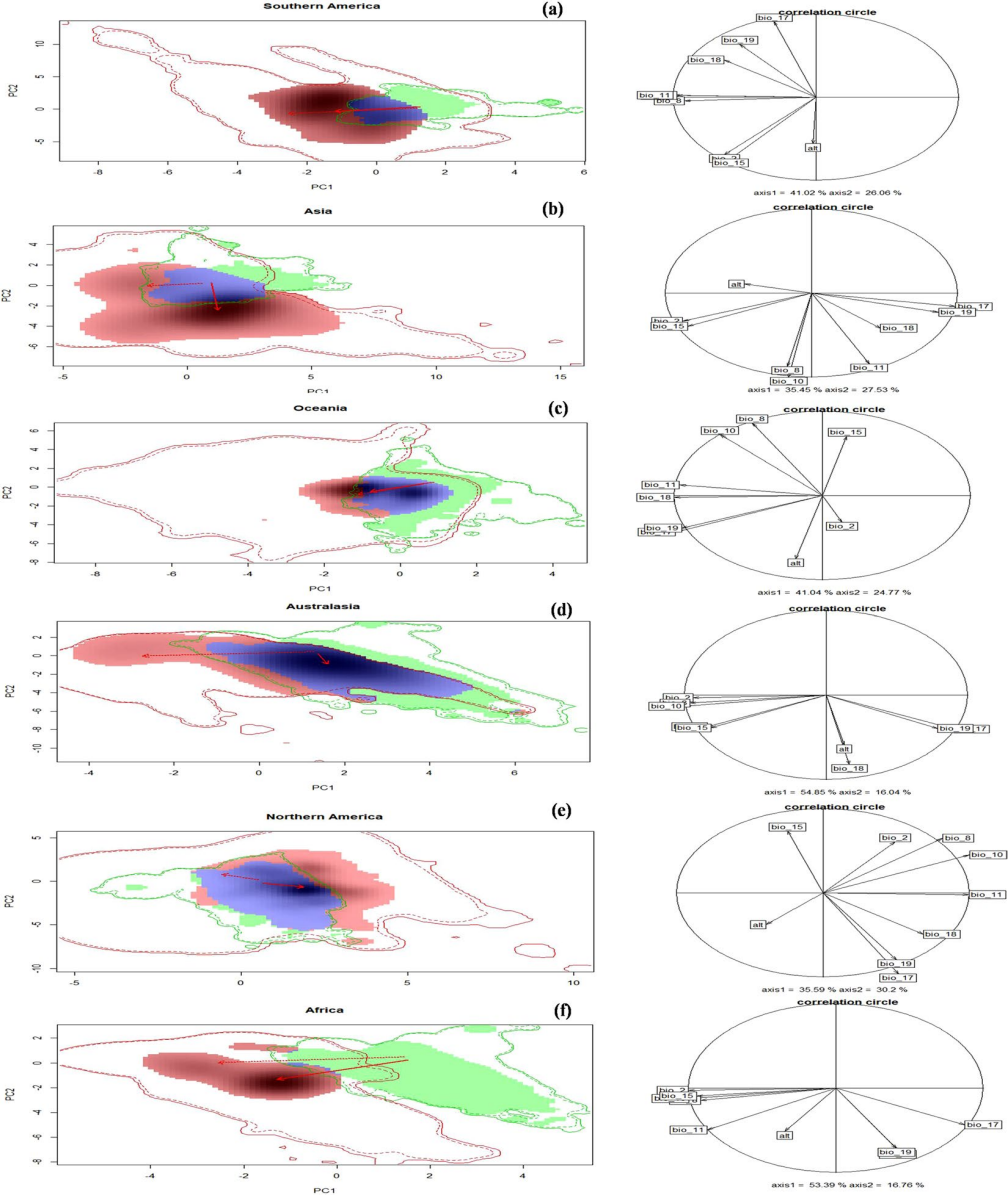
Patterns of climatic niche shift for *Leucanthemum vulgare* based on principal component analysis in different introduced regions (**a**) Southern America (**b**) Asia (**c**) Oceania (**d**) Australia (**e**) Northern America and (**f**) Africa, when compared with the native region (Europe). The first two axes of each PCA represent the density of species occurrences and the environmental space. Solid and dashed lines indicate 100% and 90% of all the available (analogous and non-analogous) environments. The blue colour represents the niche overlap between native and introduced region; the green colour a combination niche unfilling (in case of intersecting (analogous)) environments and abandonment (in case of non-intersecting (non analogous)) environments and the red colour a combination of niche expansion (in case of intersecting (analogous)) environments and pioneering (in case of non-intersecting (non- analogous)) environments. The red arrow indicates the change in the niche centroid between native and introduced range. The correlation circles represent the variable importance along the first two principle axes. Figure was created in R v3.4.3^40^ (https://www.R-project.org/).

The PCA analysis showed that the principal component 1 (PC1) retained the maximum variation (in environmental variables) for all the pairwise combinations (native vs. introduced regions) ranging from 35.45% (for Asia) to 54.85% (for Australia). Whereas the maximum variation retained by the principal component 2 (PC2) ranges from 16.04% (for Australia) to 30.2% (for Northern America) (Fig. 5**;** Table 2).

**Table 2:** Variations in environmental variables between native and introduced regions of *Leucanthemum vulgare* as defined by the PCA1 and PCA2.

Regions	PCA 1 (%)	PCA 2 (%)
Native vs. Africa	53.39	16.76
Native vs. Asia	35.45	27.53
Native vs. Australia	54.85	16.04
Native vs. Northern America	35.59	30.2
Native vs. Oceania	41.04	24.77
Native vs. Southern America	41.02	26.06

Based on the classification scheme of Rodder and Engler^53^, the niche overlap between the native and introduced regions of *Leucanthemum* was very limited for Africa (Schoener’s D = 0.004), and Southern America (Schoener’s D = 0.188), low for Asia (D = 0.216) and moderate for Oceania (D = 0.417), Australia (D = 0.503) and Northern America (D = 0.518) (Table 3). Furthermore, based on the pairwise comparison between the species environmental niche in the native and introduced regions, the equivalency test was significant (p < 0.05) for Northern America, Oceania and Southern America, and non-significant (p > 0.05) for Africa, Asia and Australia, indicating non-equivalency of the climatic niches in the former and equivalency in the latter case (Table 3**)**. Contrary to this, the niche similarity tests suggested that the niches of *Leucanthemum* in Australia, Northern America and Oceania are more similar to the niche of the native region than expected by chance (p < 0.05) (Table 3**)**. These results indicated that in these three regions, *Leucanthemum* could occupy most of the areas with similar climatic condition to those found in its native range. In contrast, the climate niches in Africa, Asia and Southern America are no more similar than expected by chance (p > 0.05). This indicates that in these introduced regions the niche occupation of the species does not follow a pattern expected by native niche requirements and has likely undergone significant alterations in its climatic niche during the invasion process.

**Table 3:** Niche shift metrics based on the analogous (common) climatic spaces between the native region (Europe) and each of the introduced regions.

Region	Schoener’s D	Similarity (p value)	Equivalency (p value)	Expansion	Unfilling	Stability
Native vs. Africa	0.004	0.099	1	0.657	0.995	0.342
Native vs. Asia	0.216	0.306	1	0.247	0.038	0.752
Native vs. Australia	0.503	0.009	0.009	0.066	0.002	0.933
Native vs. Northern America	0.518	0.049	0.009	0.07	0.003	0.929
Native vs. Oceania	0.417	0.039	0.009	0.108	0.404	0.891
Native vs. Southern America	0.188	0.059	0.99	0.524	0.187	0.475

The evaluation of niche shift metrics based on the analogous (common) climatic space revealed several distinct patterns (Table 3). (i) First, the species displayed niche expansion as exhibited by Southern America and Asia i.e. higher expansion value as compared to unfilling value (Table 3). In these regions, the species occupied wide range of climatic conditions as compared to that occupied in the native region (ii) Second, it unravelled niche unfilling as exhibited by Oceania, i.e. higher unfilling value as compared to expansion value (Table 3**)**. In this case, most of the climatic space occupied by the species in the native range remained unfilled in the introduced region, which indicates that its climatic niche in Oceania is almost a subset of the native European niche (iii) Third, it showed niche stability between the native and introduced climatic space as exhibited by Australia and Northern America, i.e. higher stability values and negligible expansion and unfilling values, (Table 3). In such a case, the climatic space occupied by the species in its native and introduced regions is almost similar but not identical. (iv) Fourth, the species also showed an almost completely dislocated climatic niche in case of Africa. In such a situation, the native and introduced climatic space occupied by the species showed clear separation, i.e. both expansion and unfilling values being comparatively higher with relatively lower stability (Table 3**)**.

The visual inspection of the niche dynamic categories, by taking into consideration all the available (both analogous and non-analogous) environments between the two regions being compared, revealed that the species besides shifting its climatic niche in case of analogous (intersecting) environments also shifted its climatic niche in the non-analogous (non-intersecting) environments to a greater extent in all the pairwise comparisons (Fig. 5).

## Discussion

The present study, for the first time at a global scale, modelled the current and future distribution of *Leucanthemum* using an ensemble modelling approach. Under the current climatic conditions, the model predicted suitable habitats for invasion of this species in the southern part of Alaska, the eastern and south-western parts of Canada, parts of Mexico, the eastern part of United States of America and coastal parts of Columbia and Ecuador; the southern parts of Chile and the south-eastern part of Argentina in Southern America; almost the entire western part of Europe; the southern part of South Africa, central part of Ethiopia and Kenya and northern part of Tanzania in Africa; parts of western Himalaya in India, Pakistan and Nepal, north-western and southern parts of China and Japan in Asia and southern part of Australia and almost entire New Zealand in Australasia. The model predictions are in consonance with the current global distribution records that the species presently occurs, as either naturalized and/or invasive, and in all the continents except Antarctica^2,51^. The suitable habitat predicted by our ensemble model is wider than the present distribution of the species. This can be attributed to the fact that our model included only the abiotic factors, i.e. climatic variables. Although, three factors are known to govern the distribution of species namely: abiotic factors (A), biotic factors (B) and movement (M) – so-called “BAM diagram”^54,55^. However, such data on the biotic factors (e.g. species interactions, dispersal abilities and propagule pressure) are not readily available and their inclusion increases the complexity in modelling technique^56^. Moreover, climate is considered as the prime factor affecting species distributions at the continental and global scales as is true for the present study. Nevertheless, at the local scale, factors like substrate and biotic interactions typically become more important^13,21,57^.

Based on the results of our ensemble model, the predicted distributional maps showed that climate change would significantly influence the global distribution of *Leucanthemum*. The model suggested that there would be a gain in both the highly and moderately suitable habitats in both native and introduced regions. In particular, the areas adjacent to the current distribution range of the species are those that are under high risk of invasion. Moreover, the range of the species is expected to increase further under anthropogenic influence and climate warming. Therefore, the scientific community including the policy makers, land resource managers and other stakeholders in these High-Risk regions need to develop efficient management strategies in order to prevent the introduction, and if that fails, then to control the further spread of this invasive species. Although, our model predicts a steady increase in the potential future distribution range, however extensive shifts in suitable areas were observed only in the case of RCP 8.5 for 2070 under which the highly suitable area shows a shift towards north-western parts of Europe. Our results are in agreement with recent studies that have reported a range expansion for other invasive species under climate change^14–18,58–61^. The plausible explanation can be that the thermophilic species tend to expand their ranges with increasing temperature under climate change^62,63^. The patterns of range expansion predicted for *Leucanthemum* can be best explained with the expected changes in patterns of precipitation and increasing temperature under climate change^9^. The areas which are unsuitable for the current climate and predicted as suitable in the future might be benefited from an increase in temperature and/or precipitation during the growing summer season of the species^9,17^. Therefore, the future climate change will facilitate increased invasion of *Leucanthemum* in the suitable habitat area and as such pre-emptive management strategies need to be put in place to control its spread in such High-Risk areas.

Although species distribution models play a crucial role in predicting the potential distribution range of species under climate change, nevertheless such models are based on abiotic factors only. Biotic factors, such as inter- and intra-specific interactions, along with abiotic factors, also affect the potential range of species^64^. Therefore, the future research challenge is to incorporate the biotic (like competition, propagule pressure and dispersal ability) and other (land use and land cover changes) factors in the distribution models in order to have a more refined understanding of the species distributions under changing climate^65^.

The present study revealed Oceania to be a High-Risk region, both currently and under the future climate change scenarios. While, Northern America was at the Moderate Risk under climate change, the remaining regions were categorised as Low Risk both currently and under the climate change scenarios. These different invasion risk rankings assigned to different regions across the world under the current climatic conditions may be attributed to contrasting scenarios encountered during the early stages of invasion^31^. The initial introduction of *Leucanthemum* in to Oceania and Northern America might have been to highly suitable habitats leading to its fast spread and colonization. While as in the rest of the regions its introduction would have been into unfavourable habitats which halted its establishment and delayed the subsequent invasion phases, leaving little opportunity for the species to invade its entire climatic niche - a phenomenon referred to as colonization-lag non-equilibrium^66^. Another possible explanation may be the difference in the residence times across different regions which play an important role in the invasion process by providing time for the species to be in equilibrium with the environment in the introduced regions^67–69^. Thus, the residence time might be longer in Oceania and Northern America as compared to other regions, which is a matter of future investigation.

The occurrence of niche shift in invasive species between the native and introduced regions provide a valuable tool for the predictions of species distribution and for mechanistic understanding of invasion process^24,28,31,37^. In the present study, visual inspection of the niche dynamic categories based on all the available (both analogous and non-analogous) environments (Fig. 5) revealed that the species shifted its niche in both the analogous (unfilling and expansion) and non-analogous (abandonment and pioneering) environments. This result is important in order to understand the conceptual framework underlying invasion niche dynamics with respect to environmental availability. Till now, most of the studies that have visualized the niche dynamics based on the available climates (both analogous and non-analogous) usually describe it as unfilling and expansion which is not the real case. Instead, it actually reflects a combination of unfilling and abandonment (when occupied in the native range only) and a combination of expansion and pioneering (when occupied in the invaded range only) and should be better distinguished on the basis of nature of the environments under consideration^24,37^ (see Fig. 1). Therefore, the previous studies have resulted in misleading interpretation of potential niche shifts during biological invasions. Although the niche dynamic categories (abandonment and pioneering) unlike niche unfilling and expansion cannot be considered as true shifts in the fundamental niche of a species because they don’t result from the action of ecological processes and simply reflect the lack of opportunity in one of the regions lacking the particular type of climate^24,37^. Thus, evaluation of true niche shifts (expansion and unfilling) during the invasion process warrants clear comparison with respect to analogous environment only, so as to avoid analytical artefacts or conceptual differences and/or interpretations^32,54,55^. Also, till date, no specialized tool or package which can plot the different niche dynamic categories solely based on the analogous (intersecting) climates is available. Therefore, it remains a challenge for the future research studies, to develop such useful tools which can enhance our understandings of niche visualization in terms of plotting functions.

While calculating the niche shift metrics in terms of analogous (intersecting) climates, the present study for the first time reported the invasion niche dynamics for *Leucanthemum* across the globe. Our results, when down-scaled to the regional level, suggested that the species showed niche conservatism (niche stability) in case of Australia and Northern America as indicated by the higher stability values of 0.93 and 0.92, respectively and lower unfilling and expansion values (Table 3). These results suggest that this invasive species occupies climatic niches similar to those of its native range in these two introduced regions and such a similarity in the climatic space between the regions of origin and destination is considered to be critical factor for successful invasion in the non-native range^23,70^. In contrast, the species shifted its niche to a greater extent in Asia, Africa, Oceania and Southern America. Interestingly, these results provide evidence that *Leucanthemum* can simultaneously occupy climatic niches distinct from its native range when introduced into a new area, thereby supporting the hypothesis that same species can undergo differential invasion niche dynamics in an introduced regions^18,23,31^. Our results are in agreement with other studies which reported that invasive species may respond to the new environments in many ways, ranging from niche conservatism^27,28,71^ to complete divergence in the niches occupied in the native and introduced regions^24,26,29–31^.

The present study showed that the shift observed in the climatic niches of *Leucanthemum* in Asia and Southern America is mainly due to niche expansion. Hence, the presence of *Leucanthemum* in novel (different) environmental conditions in its introduced regions suggest that its niche has not been conserved during the process of invasion, therefore suggesting an enhanced capacity of this species to invade new regions than previously thought^31^. The plausible explanation for this expansion could be (i) availability of wide range of climates in these introduced regions. For example, Asia covers a broad range of environments, ranging from tropical to temperate climates^72^, that allows the species to occupy diverse environments. (ii) multiple introductions resulting in the enhanced genetic diversity, allowing the species to overcome the physiological limits of the native population and giving them the competitive ability to adopt to a broader range of ecological conditions^31,73^. In contrast, the niche shift of *Leucanthemum* observed in Oceania is a result of niche unfilling, and not niche expansion, implying that the species has still a lot of suitable habitat available for colonization in the future. This result is in agreement with several other studies that reported niche unfilling to be more common in invasive alien plants than niche expansion^24,28^. This process of unfilling represents a condition of imbalance between the species occupied range (i.e. actual distribution) and potential range (i.e. all areas of habitat suitability) and can be interpreted as an indication of the incomplete invasion process^72^. The possible reasons for the frequent occurrence of niche unfilling in invasive species can be attributed to recent colonization of the invaded regions, wherein the species is yet to reach the stage of equilibrium within the regional environment and hence unable to occupy all the suitable climatic space available in the novel range^24,74,75^. It can also arise due to altered biotic interactions, e.g. the lack of mutualistic partners or the presence of efficient predators, parasites, competitors or herbivores in the invaded region^24,34^. It can also be a consequence of limited species dispersal and propagule pressure^24,34,37^. So as the residence time of an invasive species in the introduced region increases and the dispersal barriers are overcome mainly due to human aided activities, unfilling may convert to stability^24^. Finally, our study also showed a clear separation of the climatic niche for this invasive species between the native and introduced regions in Africa. This can be attributed to the fact that the species has been able to occupy habitats with different climatic conditions between its native and introduced regions in Africa, as reflected by relatively higher values of both the expansion and unfilling niche dynamic categories (Table 3**)**.

Our results suggest that niche transferability may be a function of region-specific characteristic, although more intensive research efforts are needed to better resolve the association between the responses and the different biogeographical regions. Given the fact that the floral and faunal composition, and the corresponding climate conditions of an invaded landscape may be novel to the invader and heterogenous to a greater extent, it is expected that some species will show variable distributions in an introduced region and perhaps different responses in different parts of that region^76^. In case of multiple invasions by a single species in different regions, we might expect that its biotic interactions and consequent distributions to be different in each region, thereby indicating that the distributions of invasive species show a specific species-by-region interaction effect^76^. Therefore, the future research studies should focus on the idea that niche transferability as a species-specific characteristic depends upon the particular biogeographical regions under consideration. Furthermore, keeping in view the crucial role of niche shift dynamics, it is important to consider the dimension of niche transferability in studies on biological invasions^37,77^. As such, testing the niche conservatism hypothesis can guide in the selection of appropriate species distributional modelling techniques and/or methodologies in biological invasions and thereby improve invasion management strategies^34,76^.

## Conclusions

The present study, to the best of our knowledge, is the first to model the current potential distribution and predicted the future distribution of *Leucanthemum* under climate change. The study is also the first attempt to test the invasion niche dynamics downscaled to regional levels across the globe for this notorious invasive species. The current potential distribution of *Leucanthemum* coincides well with the actual distribution records, thus indicating that species distribution modelling techniques are quite useful in predicting the suitable areas for the establishment and spread of potential invaders into new areas. The present study predicted an increase in suitable habitat for potential invasion of this species under climate change. Interestingly, the species shows differential niche shift dynamics ranging from niche conservatism (Australia and Northern America) to complete divergence in the niches occupied in the native and introduced regions (Asia, Africa, Oceania and Southern America). Research findings from the present study provide a way forward in the design of powerful predictive tools for the early detection of invasion risk-prone areas and develop appropriate management strategies to prevent the establishment and further spread of this invasive species across the globe.

## Methods

### Occurrence data

The species occurrence data was collected from Global Biodiversity Information Facility database^78^ (GBIF, available at: http://www.gbif.org, accessed 25 February 2018), Centre for Agriculture and Bioscience International^79^ (CABI, available at: www.cabi.org/, accessed 25 February 2018) supplemented with several recent field and herbarium records collected by the authors. All the records were thoroughly checked for accuracy before use. A total of 133,096 occurrence points were initially recorded. As the occurrence records are often biased towards geographically convenient or environmental-friendly areas such as near cities or areas with high population density^80^, this results in the sampling bias in geographic space. Thus, spatial thinning was undertaken to remove the spatial autocorrelation and sampling bias, where in grid cells of 10 × 10 km were created and a single occurrence point was selected randomly from each cell with more than one occurrence points. After filtering, a total of 9,046 points retained, which comprised of the native European and Asia Temperate (Armenia) range (7,659 points) and the introduced regions in Asia (49 points), Africa (15 points), Australia (33 points), Oceania (83 points), Northern America (1190) and Southern America (17 points). The native and introduced regions were delineated according to biogeographical distribution scheme followed by the USDA-Germplasm Resource Information Network^81^ (GRIN, https://npgsweb.ars-grin.gov, accessed 27 February 2018).

### Climatic variables

For current climatic projections, we extracted current climate data from the WorldClim database version 1.4^80^ (available at: http://www.worldclim.org/version1), containing minima, maxima and average values of monthly, quarterly, and annual ambient temperatures as well as precipitation values recorded from 1950 to 2000. In addition, altitude was also included as one of the variables in the present study, as it has been found to be the main factor determining the distribution of the model invasive species selected for the present study^2,52^. Thus, to begin with, we included 19 climatic variables and altitude as potential predictors in the present study. All 20 selected environmental variables had a spatial resolution of 2.5 minutes (approx. ~5 km resolution at the equator) (Supplementary Table S11). Prior to modelling, we examined possible correlations between all the selected variables, since they often show high collinearity, resulting in poor model performance and misleading interpretations^82^. We performed the Pearson’s correlation analysis and selected only those variables with correlation coefficient (r^2^) < 0.75 (Supplementary Table S12). A total of nine variables were finally retained: (i) Altitude (Alt) (ii) Mean Diurnal Range (Mean of monthly (max temp − min temp)) (BIO2), (iii) Mean Temperature of Wettest Quarter (BIO8), (iv) Mean Temperature of Warmest Quarter (BIO10), (v) Mean Temperature of Coldest Quarter (BIO11), (vi) Precipitation Seasonality (Coefficient of Variation) (BIO15), (vii) Precipitation of Driest Quarter (BIO17), (viii) Precipitation of Warmest Quarter (BIO18) and (ix) BIO19 = Precipitation of Coldest Quarter (BIO19) (Table 4).

**Table 4:** Variables retained after correlation analysis to model the distribution of *Leucanthemum vulgare*.

Factors	Variables
Climatic	BIO2 = Mean Diurnal Range (Mean of monthly (max temp − min temp))
	BIO8 = Mean Temperature of Wettest Quarter
	BIO10 = Mean Temperature of Warmest Quarter
	BIO11 = Mean Temperature of Coldest Quarter
	BIO15 = Precipitation Seasonality (Coefficient of Variation)
	BIO17 = Precipitation of Driest Quarter
	BIO18 = Precipitation of Warmest Quarter
	BIO19 = Precipitation of Coldest Quarter
Topographic	Alt = Altitude

For future climatic projections, Hadley Global Environment Model 2-Atmosphere Ocean (HADGEM2-AO) representing simulations for four Representative Concentration Pathways (RCP 2.6, RCP 4.5, RCP 6.0, RCP 8.5) for the two time periods (2050 and 2070) were obtained from the fifth assessment of the Intergovernmental Panel for Climate Change^83^.

### Modelling approach

For the current and future global distribution modelling and ensemble forecasting, we used the ten statistical and machine learning models implemented in the *biomod2*^46^ package (https://CRAN.R-project.org/package=biomod2). These models include: (1) Generalised Linear Model^84^ (GLM), (2) Generalised Additive Models^85^ (GAM), (3) Multivariate Adaptive Regression Splines^86^ (MARS), (4) Generalised Boosted Models^87^ (GBM), (5) Classification Tree Analysis^88^ (CTA), (6) Flexible Discriminant Analysis^89^ (FDA), (7) Artificial Neural Networks^90^ (ANN), (8) Maximum Entropy^91^ (MAXENT), (9) Random Forest^92^ (RF) and (10) Surface Response Envelope^93^ (SRE). We used presence only records collected from various sources along with equal number of randomly generated background points for ensemble modelling and equal weightage was given to both presences and pseudo absences^94^. Although a lot of views had been put forth whether to use occurrence records from the native region, introduced regions or from the both the regions to predict the distribution of invasive species. The most commonly used framework is to use combined occurrence records from both the native and introduced regions simultaneously, owing to the inherent advantage that by using distribution data from the native range, it will make use of those occurrence records that are likely to be in equilibrium with the regional environment there in addition to including records from the introduced regions which may provide additional information about the expansion of the realized niche in the novel ranges^95–97^. Therefore, for the present study, we built the ensemble model based on the combined occurrence records from both the native and introduced regions of the species. We evaluated the predictive performance of each model using a repeated split sampling in which the models were calibrated with 70% of occurrence data and evaluated for the remaining 30%. The area under the curve (AUC) of the receiver operating characteristics (ROC), true skills statistics (TSS) and Cohen’s KAPPA were used to estimate the performance of the model^98–101^. AUC is a threshold independent measure of model evaluation that ranges from 0–1. An AUC value between 0.5 and 0.7 indicates poor model performance, 0.7–0.9 indicates good performance, and >0.9 indicated high performance^59,99^. While as both TSS and KAPPA are threshold dependent metrics of model evaluation and range from −1 to +1. Generally, the values of TSS and KAPPA below 0.40 indicates poor model performance, values ranging between 0.40 to 0.75 specifies good model performance and the values above 0.75 indicates excellent model performance^100,101^. We generated a total of nine ensemble maps including: one current and eight future maps corresponding to four representative concentration pathways (RCPs 2.6, 4.5, 6.0 and 8.5) for two time periods (i.e. 2050 and 2070). We constructed ensemble maps based on the median of three runs of all the selected models in which individual model had AUC value equal to or greater than 0.7 and TSS and Kappa values equal to or greater than 0.6 based on current climatic scenario^41,60,102^ (Supplementary Table S13). The median files obtained were used for further analysis.

### Area change analysis

We analysed the change in habitat suitability for the two time periods i.e., 2050 (average for 2041–2060) and 2070 (average for 2061–2080) for the four RCPs: 2.6, 4.5, 6.0 and 8.5. We categorised the final ensemble model into four categories following Hamid *et al*.^103^ and Ahmad *et al*.^104^ as: Not suitable (0.0–0.25) coded as blue colour, Low suitable (0.25–0.50) coded as white, Moderately suitable (0.50–0.75) coded as light red and Highly suitable (0.75–1.00) coded as red colour. We calculated the percentage of area change between two SDMs (current vs future) by simple differencing using DIVA-GIS software^105^.

Further to examine the distributional change between current and future climate change scenarios, we compared the current and future habitat suitability maps using SDM toolbox v2.2b^106^ (available from http://sdmtoolbox.org/downloads). It is a python-based GIS toolkit that compares the distributional changes between two binary SDMs (current and future) and generates an output raster depicting four categories: (i) range expansion (present in the future SDM only), (ii) no occupancy (absent in both SDMs), (iii) no change (present in both) (iv) range contraction (present under current SDM only).

### Invasion risk categorisation (present and future)

We calculated invasion risk at the regional level by using the final ensemble models. For this, we clipped the final ensemble models to different biogeographical regions. For each region, we categorised the ensemble model into four zones i.e., no risk (0.00–0.25), low risk (0.25–0.50), moderate risk (0.50–0.75) and high risk (0.75–1.00) and obtained percentage of high risk invasion by using simple differencing between High Risk zone i.e., (0.75–1.00) and total area of land in Km^2^. We further classified the regions at high-risk (>30% of the total area prone to high risk of invasion by the species), moderate risk (>10% to 30% of the total area prone to high risk of invasion) and low risk (<or =10% of the total area prone to high risk of invasion).

### Niche shift test

In the present study, we analysed the niche shifts between native region (Europe) and each of the introduced regions (Asia, Africa, Australia, Northern America, Oceania and Southern America) separately. We adopted the modified principal component analysis (PCA-env) approach implemented in the *Ecospat* package version 2.1.1^107^. First, based on the observed occurrences of the species, the environmental space of the selected environmental variables was transformed into a two-dimensional space defined by the first and second principal components^108^. The two-dimensional space was then divided into 100 × 100 grid cells bounded by the minimum and maximum PCA values in the background data. Then by applying the kernel density function, we converted the occurrence records of both the ranges into smoothed densities or occurrences. This method was preferred because of its robustness and the kernel density metric used in the method allows for the unbiased comparisons of occurrence densities when the environments are not equally available^28,31^. We also generated the 10000 random background points for the native and each of the introduced continents separately to have an account of the available (background) environments. We performed the niche equivalency and niche similarity test based on the 95% confidence interval to test the null hypothesis of the similar and equivalent niches of *Leucanthemum* in its native and introduced regions^108^. We used Schoener’s D metrics a measure of niche overlap that ranges from 0 (no overlap) to 1 (complete overlap).

We also calculated three niche shift metrics namely: niche unfilling (U), stability (S) and expansion (E) by adopting the Centroid, Overlap, Unfilling, Expansion (COUE) framework^37^ to provide a more complete picture of niche dynamics. Since these niche dynamic categories reflect a case of ‘analogue’ niche space, which is defined as region of climate space shared between the native and invaded range of a particular invasive species^24,67^, therefore in the present study, we calculated these niche shift metrics (i.e. expansion, stability and unfilling) by considering only the analogous (common) environmental conditions between the two regions being compared, using the function *ecospat*.*niche*.*dyn*.*index()* implemented in the *ecospat*^107^ package. This approach was aimed to help distinguish between true niche shift (unfilling and expansion) and shift caused due to the availability of climates in only one of the two regions being compared (abandonment and pioneering)^24,32^. Niche unfilling means the environmental conditions colonized by a species in its native range but not in the invaded range despite the presence of similar environmental conditions in the latter. Niche stability means the environmental conditions colonized by a species in both the native and invaded range, while as niche expansion means environmental conditions colonized by a species in its invaded range but not in its native range in spite of the presence of similar environmental conditions in the latter^24,32,37^. Further, in case of analogous (common) environments also, a particular climatic condition common in one of the regions may be less frequent in the other. These less frequent or marginal climatic conditions also matters when quantifying niche dynamics between two ranges^37^. Therefore, following the recommendations of Guisan *et al*.^37^ and Di cola *et al*.^107^, the present study quantified the niche shift metrics by taking into account only 90% of the most common environments shared between the two regions after eliminating 10% of the marginal environments. Additionally, we also visualized the niche dynamics by taking into account all the available (analogous and non-analogous) climates between the two regions being compared in order to see if the species has shifted its niche into the non‐analogous climatic space as well.

## Supplementary information

Supplementary info

## Data Availability

The occurrence records for *Leucanthemum vulgare* are available from GBIF: http://www.gbif.org; CABI: www.cabi.org/. All climate data layers are available as raster grids from the WorldClim database version 1.4: http://www.worldclim.org/version1.
