# Supplementary material for: Global distribution modelling, invasion risk assessment and niche dynamics of Leucanthemum vulgare (Ox-eye Daisy) under climate change
Source: Sci Rep. 2019 Aug 6;9:11395. doi: 10.1038/s41598-019-47859-1 (PMC6684661; doi:10.1038/s41598-019-47859-1)
Supplement: Supplementary file 1 — Supplementary info [file 41598_2019_47859_MOESM1_ESM.pdf]

Global distribution modelling, invasion risk assessment and niche dynamics of *Leucanthemum vulgare* (Ox-eye Daisy) under climate change

Rameez Ahmad[1], *Anzar A. Khuroo*[1*], *Bipin Charles*[2], *Maroof Hamid*[1], *Irfan Rashid*[3], *N. A. Aravind*[2]

[1]Centre for Biodiversity & Taxonomy, Department of Botany

University of Kashmir, Srinagar – 190006, J & K, India

[2]Ashoka Trust for Research in Ecology and the Environment (ATREE),

Royal Enclave, Srirampura, Jakkur PO, Bengaluru – 560064, India

[3]Biological Invasions Laboratory, Department of Botany

University of Kashmir, Srinagar – 190006, J & K, India

[*]Corresponding author e-mail: anzarak@gmail.com

Supplementary information

Supplementary Table S1: AUC (ROC), TSS and KAPPA values of ensemble model

	Testing data	Cut off	Sensitivity	Specificity
**TSS**	0.850	445.0	95.754	89.225
**ROC**	0.977	438.5	95.799	89.225
**KAPPA**	0.845	46.0	99.094	81.370

Supplementary Table S2: Regional invasion risk categorization in terms of the area (km^2) invaded by *Leucanthemum vulgare* under current climatic conditions.

Category	Southern America	Oceania	Northern America	Australia	Asia	Africa
**No Risk** (0 - 0.25)	12,753,989	111,387	26,523,996	5,805,395	48,378,451	23,818,782
**Low Risk** (0.25 - 0.5)	1,017,237	6,901	2,745,450	323,734	1,940,117	329,071
**Moderate Risk** (0.5 -0.75)	563,076	51,452	2,834,536	177,547	554,858	84,343
**High Risk** (0.75 - 1)	234,038	214,258	2,636,435	263,849	280,962	8466
Total Area (Sq. km)	14,568,340	383,998	34,740,417	6,570,525	51,154,388	24,240,662
**Invaded Area (%age)**	2%	56%	8%	4%	1%	0.03%
**Risk Category**	**Low Risk**	**High Risk**	**Medium Risk**	**Low Risk**	**Low Risk**	**Low Risk**

Supplementary Table S3: Regional invasion risk categorization in terms of the area (km^2) invaded by *Leucanthemum vulgare* under RCP 2.6 2050.

Category	Southern America	Oceania	Northern America	Australia	Asia	Africa
**No Risk** (0 - 0.25)	13,594,190	113,808	27,148,802	6,173,220	49,748,837	24,168,654
**Low Risk** (0.25 - 0.5)	229,345	6,934	1,582,092	81,758	438,646	36,909
**Moderate Risk** (0.5 - 0.75)	322,203	32,051	1,861,275	81,247	338,607	26,928
**High Risk** (0.75 - 1)	422,604	231,206	4,148,315	234,286	628,281	8,186
Total Area	14,568,342	383,999	34,740,484	6,570,511	51,154,371	24,240,677

(Sq. km)						
**Invaded Area (%age)**	3%	60%	12%	4%	1%	0.03%
**Risk Category**	**Low Risk**	**High Risk**	**Medium Risk**	**Low Risk**	**Low Risk**	**Low Risk**

Supplementary Table S4: Regional invasion risk categorization in terms of the area (km^2) invaded by *Leucanthemum vulgare* under RCP 2.6 2070.

**Category**	**Southern America**	**Oceania**	**Northern America**	**Australia**	**Asia**	**Africa**
**No Risk** (0 - 0.25)	13,624,066	113,808	27,529,029	6,196,558	49,700,086	24,170,170
**Low Risk** (0.25 - 0.5)	213,072	4,645	1,352,072	75,828	469,016	38,787
**Moderate Risk** (0.5 - 0.75)	310,970	26,303	1,921,209	93,039	395,181	23,684
**High Risk** (0.75 - 1)	420,232	239,243	3,938,175	205,084	590,087	8,037
Total Area (Sq. km)	14,568,340	383,999	34,740,485	6,570,509	51,154,370	24,240,678
**Invaded Area (%age)**	3%	62%	11%	3%	1%	0.03%
**Risk Category**	**Low Risk**	**High Risk**	**Medium Risk**	**Low Risk**	**Low Risk**	**Low Risk**

Supplementary Table S5: Regional invasion risk categorization in terms of the area (km^2) invaded by *Leucanthemum vulgare* under RCP 4.5 2050.

**Category**	**Southern America**	**Oceania**	**Northern America**	**Australia**	**Asia**	**Africa**
**No Risk** (0 - 0.25)	13,683,243	121,087	27,067,638	6,257,118	49,634,470	24,200,030
**Low Risk** (0.25 - 0.5)	181,779	13,654	1,773,819	70,080	516,120	25,594
**Moderate Risk** (0.5 - 0.75)	237,580	34,966	1,960,111	76,042	351,898	11,397
**High Risk** (0.75 - 1)	465,739	214,291	3,938,916	167,269	651,883	3,656
Total Area (Sq. km)	14,568,341	383,998	34,740,484	6,570,509	51,154,371	24,240,677
**Invaded Area (%age)**	3%	56%	11%	3%	1%	0.02%
**Risk Category**	**Low Risk**	**High Risk**	**Medium Risk**	**Low Risk**	**Low Risk**	**Low Risk**

Supplementary Table S6: Regional invasion risk categorization in terms of the area (km^2) invaded by *Leucanthemum vulgare* under RCP 4.5 2070.

Category	Southern America	Oceania	Northern America	Australia	Asia	Africa
**No Risk** (0 - 0.25)	13,721,602	125,600	26,378,105	6,270,870	49,195,165	24,206,470
**Low Risk** (0.25 - 0.5)	158,820	11,562	1,399,604	81,230	756,912	19,830
**Moderate Risk** (0.5 - 0.75)	246,259	36,662	2,695,595	68,960	369,208	11,068
**High Risk** (0.75 - 1)	441,660	210,174	4,267,179	149,449	833,086	3,310
Total Area (Sq. km)	14,568,341	383,998	34,740,483	6,570,509	51,154,371	24,240,678
**Invaded Area (%age)**	3%	55%	12%	2%	2%	0.01%
**Risk Category**	**Low Risk**	**High Risk**	**Medium Risk**	**Low Risk**	**Low Risk**	**Low Risk**

Supplementary Table S7: Regional invasion risk categorization in terms of the area (km^2) invaded by *Leucanthemum vulgare* under RCP 6.0 2050.

Category	Southern America	Oceania	Northern America	Australia	Asia	Africa
**No Risk** (0 - 0.25)	13,645,840	116,838	27,201,671	6,223,453	49,608,859	24,167,716
**Low Risk** (0.25 - 0.5)	213,402	11,282	1,547,505	65,336	488,599	36,135
**Moderate Risk** (0.5 - 0.75)	304,547	30,190	1,957,328	89,761	375,747	26,006
**High Risk** (0.75 - 1)	404,553	225,688	4,033,981	191,958	681,166	10,821
Total Area (Sq. km)	14,568,342	383,998	34,740,485	6,570,508	51,154,371	24,240,678
**Invaded Area (%age)**	3%	59%	12%	3%	1%	0.04%
**Risk Category**	**Low Risk**	**High Risk**	**Medium Risk**	**Low Risk**	**Low Risk**	**Low Risk**

Supplementary Table S8: Regional invasion risk categorization in terms of the area (km^2) invaded by *Leucanthemum vulgare* under RCP 6.0 2070.

Category	Southern America	Oceania	Northern America	Australia	Asia	Africa
**No Risk** (0 - 0.25)	13,666,444	118,090	26,691,842	6,261,005	49,572,477	24,201,825
**Low Risk** (0.25 - 0.5)	215,263	10,162	1,794,802	67,214	554,067	22,959
**Moderate Risk** (0.5 - 0.75)	280,468	44,947	2,073,606	85,809	342,757	`12,040
**High Risk** (0.75 - 1)	406,167	210,800	4,180,234	156,481	685,070	3854
Total Area (Sq. km)	14,568,342	383,999	34,740,484	6,570,509	51,154,371	24,240,678
**Invaded Area (%age)**	3%	55%	12%	2%	1%	0.02%
**Risk Category**	**Low Risk**	**High Risk**	**Medium Risk**	**Low Risk**	**Low Risk**	**Low Risk**

Supplementary Table S9: Regional invasion risk categorization in terms of the area (km^2) invaded by *Leucanthemum vulgare* under RCP 8.5 2050.

Category	Southern America	Oceania	Northern America	Australia	Asia	Africa
**No Risk (0 - 0.25)**	13,694,920	118,633	26,800,775	6,241,998	49,687,141	24,196,143
**Low Risk (0.25 - 0.5)**	186,210	11,710	1,497,222	72,419	460,896	26,105
**Moderate Risk (0.5 - 0.75)**	274,225	38,227	2,012,519	80,159	282,477	13,588
**High Risk (0.75 - 1)**	412,985	215,428	4,429,969	175,933	723,856	4,842
**Total Area (Sq. km)**	14,568,340	383,998	34,740,485	6,570,509	51,154,370	24,240,678
**Invaded Area (%age)**	3%	56%	13%	3%	1%	0.02%
**Risk Category**	**Low Risk**	**High Risk**	**Medium Risk**	**Low Risk**	**Low Risk**	**Low Risk**

Supplementary Table S10: Regional invasion risk categorization in terms of the area (km^2) invaded by *Leucanthemum vulgare* under RCP 8.5 2070.

Category	Southern America	Oceania	Northern America	Australia	Asia	Africa
**No Risk** (0 - 0.25)	13,811,264	133,983	26,070,215	6,327,889	48,880,259	24,222,231
**Low Risk** (0.25 - 0.5)	166,660	22,416	1,388,668	62,092	693,914	11,249
**Moderate Risk** (0.5 - 0.75)	195,960	50,925	2,339,201	59,868	558,135	5,880
**High Risk** (0.75 - 1)	394,456	176,674	4,942,400	120,659	1,022,062	1318
Total Area (Sq. km)	14,568,340	383,998	34,740,484	6,570,508	51,154,370	24,240,678
**Invaded Area (%age)**	3%	46%	14%	2%	2%	0.01%
**Risk Category**	**Low Risk**	**High Risk**	**Medium Risk**	**Low Risk**	**Low Risk**	**Low Risk**

Supplementary Table S11: Climatic and topographic variables used to model the distribution of *Leucanthemum vulgare* during the present study. Variables in bold indicate the ones retained after correlation analysis to build the model.

Factors	Variables
Climatic	BIO1 = Annual Mean Temperature
	**BIO2 = Mean Diurnal Range (Mean of monthly (max temp - min temp))**
	BIO3 = Isothermality (BIO2/BIO7) (* 100)
	BIO4 = Temperature Seasonality (standard deviation *100)
	BIO5 = Max Temperature of Warmest Month
	BIO6 = Min Temperature of Coldest Month
	BIO7 = Temperature Annual Range (BIO5-BIO6)
	**BIO8 = Mean Temperature of Wettest Quarter**
	BIO9 = Mean Temperature of Driest Quarter
	**BIO10 = Mean Temperature of Warmest Quarter**
	**BIO11 = Mean Temperature of Coldest Quarter**
	BIO12 = Annual Precipitation
	BIO13 = Precipitation of Wettest Month

	BIO14 = Precipitation of Driest Month
	**BIO15 = Precipitation Seasonality (Coefficient of Variation)**
	BIO16 = Precipitation of Wettest Quarter
	**BIO17 = Precipitation of Driest Quarter**
	BIO18 = Precipitation of Warmest Quarter
	**BIO19 = Precipitation of Coldest Quarter**
Topographic	**Altitude**

Supplementary Table S12: Pairwise correlation coefficients between variables. Red colour indicates the variables that are correlated and excluded from the present study

	Alt	*bio_1*	*bio_2*	*bio_3*	*bio_4*	*bio_5*	*bio_6*	*bio_7*	*bio_8*	*bio_9*	*bio_10*	*bio_11*	*bio_12*	*bio_13*	*bio_14*	*bio_15*	*Bio_16*	*Bio_17*	*bio_18*	*bio_19*
Alt	1																			
bio_1	-0.05																			
bio_2	0.43	0.14																		
bio_3	0.36	0.69	0.36																	
bio_4	-0.07	-0.63	0.34	-0.71																
bio_5	0.01	0.60	0.71	0.29	0.18															
bio_6	-0.06	**0.87**	-0.23	0.69	**-0.90**	0.17														
bio_7	0.06	-0.52	0.58	-0.51	**0.95**	0.34	**-0.86**													
bio_8	-0.18	0.11	0.16	-0.21	0.35	0.40	-0.12	0.32												
bio_9	0.09	**0.75**	0.02	0.72	**-0.76**	0.26	**0.81**	-0.64	-0.39											
bio_10	-0.15	**0.76**	0.46	0.29	0.01	**0.93**	0.37	0.11	0.43	0.36										
bio_11	-0.01	**0.93**	-0.07	**0.76**	**-0.87**	0.29	**0.98**	**-0.78**	-0.10	**0.84**	0.48									
bio_12	0.23	0.30	0.04	0.44	-0.40	-0.004	0.34	-0.33	-0.35	0.43	0.04	0.37								
bio_13	0.30	0.23	0.14	0.43	-0.30	0.04	0.24	-0.20	-0.28	0.37	0.04	0.28	**0.90**							
bio_14	0.06	0.24	-0.13	0.25	-0.35	-0.12	0.31	-0.36	-0.27	0.28	-0.01	0.30	0.72	0.41						
bio_15	0.25	-0.11	0.32	0.08	0.17	0.13	-0.21	0.27	0.01	-0.03	0.01	-0.14	0.04	0.41	-0.54					
bio_16	0.28	0.24	0.11	0.43	-0.32	0.02	0.26	-0.23	-0.30	0.38	0.03	0.30	**0.92**	**0.99**	0.44	0.38				
bio_17	0.10	0.29	-0.09	0.31	-0.39	-0.06	0.35	-0.37	-0.30	0.35	0.03	0.35	**0.77**	0.47	**0.98**	-0.51	0.50			
bio_18	0.21	0.01	0.03	0.03	0.03	-0.03	-0.05	0.02	0.19	-0.17	0.01	-0.02	0.58	0.48	0.60	-0.08	0.48	0.59		
bio_19	0.14	0.30	0.01	0.49	-0.49	-0.04	0.41	-0.41	-0.52	0.58	-0.02	0.42	**0.87**	**0.81**	0.53	0.10	**0.84**	0.59	0.15	1

Supplementary Table S13: TSS, AUC (ROC) and KAPPA values for individual models

**GLM, RUN1**				
	Testing.data	Cutoff	Sensitivity	Specificity
TSS	0.810	463	94.491	86.577
ROC	0.951	486	94.264	86.913
KAPPA	0.795	74	98.453	77.852
**GBM, RUN1**				
	Testing.data	Cutoff	Sensitivity	Specificity
TSS	0.817	370	95.434	86.242
ROC	0.954	355	95.472	86.242
KAPPA	0.796	40	98.868	75.839
**GAM, RUN1**				
	Testing.data	Cutoff	Sensitivity	Specificity
TSS	0.814	598	93.208	88.255
ROC	0.954	596	93.208	88.255
KAPPA	0.811	35	98.906	77.517
**CTA, RUN1**				
	Testing.data	Cutoff	Sensitivity	Specificity
TSS	0.802	506.5	93.925	86.242
ROC	0.911	507.5	93.925	86.242
KAPPA	0.686	171.5	94.302	85.235
**ANN, RUN1**				
	Testing.data	Cutoff	Sensitivity	Specificity
TSS	0.700	541.0	94.302	71.812
ROC	0.900	545.5	85.509	84.564
KAPPA	0.699	102.0	97.358	70.805
**SRE, RUN1**				
	Testing.data	Cutoff	Sensitivity	Specificity
TSS	0.688	495	76.83	91.946
ROC	0.844	500	76.83	91.946
KAPPA	0.366	495	76.83	91.946
**FDA, RUN1**				
	Testing.data	Cutoff	Sensitivity	Specificity
TSS	0.789	747.0	97.170	81.879
ROC	0.942	757.5	94.113	85.235
KAPPA	0.810	677.0	99.170	75.839
**MARS, RUN1**				
	Testing.data	Cutoff	Sensitivity	Specificity
TSS	0.803	334.0	94.755	85.570
ROC	0.952	326.5	94.906	85.570
KAPPA	0.781	70.0	98.453	75.839
**RF, RUN1**				

	Testing.data	Cutoff	Sensitivity	Specificity
TSS	0.856	889	94.717	90.940
ROC	0.963	888	94.717	90.940
KAPPA	0.845	313	98.604	84.899
**MAXENT Philips, RUN1**				
	Testing.data	Cutoff	Sensitivity	Specificity
TSS	0.720	10.0	84.151	87.248
ROC	0.874	67.5	82.566	89.597
KAPPA	0.486	0.0	100.000	0.000
**GLM, RUN2**				
	Testing.data	Cutoff	Sensitivity	Specificity
TSS	0.823	269.5	96.717	85.570
ROC	0.959	258.5	96.868	85.570
KAPPA	0.829	75.0	98.868	80.201
**GBM, RUN2**				
	Testing.data	Cutoff	Sensitivity	Specificity
TSS	0.817	541.0	93.774	87.919
ROC	0.961	497.5	94.113	87.584
KAPPA	0.822	39.0	98.943	79.530
**GAM, RUN2**				
	Testing.data	Cutoff	Sensitivity	Specificity
TSS	0.818	409.0	95.509	86.242
ROC	0.954	400.5	95.547	86.242
KAPPA	0.822	46.0	98.981	78.859
**CTA, RUN2**				
	Testing.data	Cutoff	Sensitivity	Specificity
TSS	0.849	134	95.925	88.926
ROC	0.941	111	95.962	88.926
KAPPA	0.775	59	97.887	78.188
**ANN, RUN2**				
	Testing.data	Cutoff	Sensitivity	Specificity
TSS	0.729	633.0	91.396	81.544
ROC	0.924	629.5	91.509	81.544
KAPPA	0.685	57.0	97.925	65.772
**SRE, RUN2**				
	Testing.data	Cutoff	Sensitivity	Specificity
TSS	0.682	495	77.547	90.604
ROC	0.841	500	77.547	90.604
KAPPA	0.370	495	77.547	90.604
**FDA, RUN2**				
	Testing.data	Cutoff	Sensitivity	Specificity
TSS	0.811	737.0	98.000	82.886
ROC	0.948	749.5	96.604	84.899

KAPPA	0.816	722.0	98.868	78.523
**MARS, RUN2**				
Testing.data	Cutoff	Sensitivity	Specificity	
TSS	0.810	301.5	95.094	85.906
ROC	0.959	290.5	95.245	85.906
KAPPA	0.812	89.0	98.491	80.537
**RF, RUN2**				
Testing.data	Cutoff	Sensitivity	Specificity	
TSS	0.874	786	96.755	90.604
ROC	0.969	824	96.453	90.940
KAPPA	0.852	383	98.340	87.919
**MAXENT Philips, RUN2**				
Testing.data	Cutoff	Sensitivity	Specificity	
TSS	0.748	51.0	84.000	90.604
ROC	0.894	51.5	83.849	90.940
KAPPA	0.537	0.0	100.000	0.000
**GLM, RUN3**				
Testing.data	Cutoff	Sensitivity	Specificity	
TSS	0.837	428.0	94.075	89.597
ROC	0.965	426.5	94.113	89.597
KAPPA	0.806	104.0	98.000	82.886
**GBM, RUN3**				
Testing.data	Cutoff	Sensitivity	Specificity	
TSS	0.843	476.0	94.113	89.933
ROC	0.968	476.5	94.075	90.268
KAPPA	0.799	67.0	97.774	83.221
**GAM, RUN3**				
Testing.data	Cutoff	Sensitivity	Specificity	
TSS	0.841	430	94.528	89.262
ROC	0.964	433	94.528	89.597
KAPPA	0.826	36	99.057	78.859
**CTA, RUN3**				
Testing.data	Cutoff	Sensitivity	Specificity	
TSS	0.839	452	94.302	89.597
ROC	0.934	452	94.302	89.597
KAPPA	0.739	70	96.377	82.215
**ANN, RUN3**				
Testing.data	Cutoff	Sensitivity	Specificity	
TSS	0.688	570.5	97.019	71.812
ROC	0.848	571.0	97.019	71.812
KAPPA	0.693	570.5	97.019	71.812
**SRE, RUN3**				
Testing.data	Cutoff	Sensitivity	Specificity	

TSS	0.711	495	76.491	94.631
ROC	0.856	500	76.491	94.631
KAPPA	0.374	495	76.491	94.631
**FDA, RUN3**				
Testing.data	Cutoff	Sensitivity	Specificity	
TSS	0.816	754.0	96.000	85.570
ROC	0.956	756.5	94.755	87.584
KAPPA	0.787	677.0	98.642	75.839
**MARS, RUN3**				
Testing.data	Cutoff	Sensitivity	Specificity	
TSS	0.831	496.0	93.132	89.933
ROC	0.966	496.5	93.132	89.933
KAPPA	0.794	69.0	98.075	80.537
**RF, RUN3**				
Testing.data	Cutoff	Sensitivity	Specificity	
TSS	0.883	829	95.396	92.953
ROC	0.974	824	95.472	92.953
KAPPA	0.849	39	98.981	82.550
**MAXENT Philips, RUN3**				
Testing.data	Cutoff	Sensitivity	Specificity	
TSS	0.773	20	88.792	88.255
ROC	0.907	26	88.491	88.926
KAPPA	0.624	0	100.000	0.000
